# Identifying the Aetiology of Acute Liver Failure Is Crucial to Impact Positively on Outcome

**DOI:** 10.3390/children10040733

**Published:** 2023-04-16

**Authors:** Angelo Di Giorgio, Silvia Gamba, Naire Sansotta, Emanuele Nicastro, Michele Colledan, Lorenzo D’Antiga

**Affiliations:** 1 Paediatric Hepatology, Gastroenterology and Transplantation Department, ASST Hospital Papa Giovanni XXIII Bergamo, Piazza OMS 1, 24127 Bergamo, Italy; 2 Department of Organ Failure and Transplantation, Surgery University Milano-Bicocca, ASST Hospital Papa Giovanni XXIII, 24127 Bergamo, Italy

**Keywords:** acute liver failure, children, liver transplantation, aetiology

## Abstract

Management of children with acute liver failure is challenging. In this retrospective study, paediatric patients diagnosed with ALF at our centre, in the last 26 years, were divided into two groups (G1 = diagnosed from 1997 to 2009; G2 = from 2010 to 2022) and compared to see whether they differed with regard to aetiologies, need for liver transplantation (LT), and outcome. A total of 90 children (median age 4.6 years, range 1.2–10.4; M/F = 43/47) were diagnosed with ALF, by autoimmune hepatitis (AIH) in 16 (18%), paracetamol overdose in 10 (11%), Wilson disease in 8 (9%), and other causes in 19 (21%); 37 (41%) had indeterminate ALF (ID-ALF). Comparing the two periods, the clinical features, aetiologies, and median peak values of INR [3.8 (2.9–4.8) in G1 vs. 3.2 (2.4–4.8) in G2] were similar (*p* > 0.05). The percentage of ID-ALF tended to be higher in G1 compared to G2 (50% vs. 32% in G2, *p* = 0.09). The overall percentage of patients diagnosed with Wilson disease, inborn errors of metabolism, neonatal hemochromatosis or viral infection was higher in G2 (34% vs. 13% in G1, *p* = 0.02). A total of 21/90 patients (23%; 5 with indeterminate ALF) were treated with steroids; 12 (14%) required extracorporeal liver support treatment. The need for LT was significantly higher in G1 compared to G2 (56% vs. 34%; *p* = 0.032). Among 37 children with ID-ALF, 6 (16%) developed aplastic anaemia (all in G2, *p* < 0.001). The survival rate at last follow up was of 94%. On a KM curve, the transplant-free survival was lower in G1 compared to G2. In conclusion, we report a lower need for LT in children diagnosed with PALF during the most recent period compared to the first era. This suggests improvements over time in the diagnosis and management of children with PALF.

## 1. Introduction

Acute liver failure (ALF) indicates a loss of liver function that occurs when many cells in the liver die or become very damaged in a short amount of time.

In paediatrics, ALF is defined by the biochemical features of acute liver injury, coagulopathy, and no evidence of pre-existing chronic liver disease [1,2]. The management of neonates, infants and children with ALF is challenging because there is often a very short time window to identify the aetiology, start a specific treatment, and decide whether liver transplantation (LT) is needed in an individual patient.

In such scenarios, identifying the aetiology of ALF is crucial to quickly initiate disease-specific therapies in treatable disorders. Unfortunately, the aetiology of ALF often remains unknown in approximately 50% of cases, which end up with a diagnosis of indeterminate ALF (ID-ALF) [3,4]. This percentage is likely overestimated because an extensive diagnostic work-up is not always carried out, for instance, in the case of missed investigation of autoimmunity markers and/or incomplete metabolic screening [5].

In Italy, the Liver Disease Working Group of the Italian Society for Gastroenterology, Hepatology and Nutrition (SIGENP) has published a position paper to provide recommendations on the diagnostic work-up that should be performed in all paediatric patients diagnosed with ALF [6]. 

Of interest, previous studies have demonstrated that in recent years there have been improvements in the management of children with ALF [6,7,8,9]. Authors have reported that children with ALF due to autoimmune hepatitis (AIH) may have a high transplant free-survival rate when diagnosed and treated promptly [6,10,11,12,13].

Our institution is a national referral centre for paediatric hepatology; the transplant program started in 1997, and since then we have diagnosed and treated several children with ALF. 

In this study, all children diagnosed with ALF were divided into two eras and compared to see whether they differed mainly with regard to aetiology, transplant-free survival, and final outcome.

## 2. Materials and Methods

### 2.1. Patient Selection 

Patients were retrospectively selected from those referred to Paediatric Liver, Gastrointestinal, and Transplantation Unit of the Hospital Papa Giovanni XXIII, Bergamo, between 1997 and 2022. In this period, 809 paediatric liver transplants (79% from split livers) have been performed at our institution. Data on clinical features, laboratory, radiology, histology, treatment and outcome were collected and analysed.

### 2.2. Diagnosis and Management 

According to the Paediatric Acute Liver Failure (PALF) Study Group, the diagnosis of ALF is based on (1) biochemical evidence of acute liver injury within 8 weeks of the onset of illness; (2) the presence of coagulopathy (INR ≥ 2.0) regardless of HE; and (3) no previously recognised chronic liver disease [1].

The general assessment, the diagnostic work up, and the medical treatment of children with ALF due to autoimmune hepatitis (AIH) or Wilson disease (WD) were performed according to the protocols used at our Liver Unit, as reported in our previous studies [13,14]. Autoimmune hepatitis (AIH) was classified as type 1 (positivity for smooth muscle, ASMA, and/or anti-nuclear, ANA) and type 2 (positivity for liver/kidney microsomal antibody, LKM-1, and/or liver citosol antibody, (LC1) [9].

If no aetiology was identified, the patients were considered affected by indeterminate ALF (ID-ALF).

Hepatic encephalopathy (HE). HE was evaluated on physical examination and by electroencephalogram (EEG) in accordance with its standard classification.

Physical examination

Grade I—alert, mood changes, slow mentation.

Grade II—lethargic, confused.

Grade III—stuporous, obeys simple commands.

Grade IV—unarousable, increased or flaccid muscle tone, hyper-reflexic extensive plantar response or absent reflexes.

EEG changes:

Grade I: low-frequency, frontalised alpha activity with random theta waves.

Grade II: predominant theta activity with random delta waves. 

Grade III: high voltage delta–theta activity with triphasic waves.

Grade IV: the EEG tends to flatten [15,16].

Histological evaluation. A liver biopsy was performed if (a) no specific aetiology of ALF was identified, despite an extensive diagnostic work up; or (b) the suspected aetiology was AIH. The procedure was performed in the intraoperative room, under conscious sedation, and after fresh frozen plasma (FFP) infusion to at least partially correct the coagulopathy. A retrospective evaluation of histology samples had already been performed and reported in our previous studies [13,17].

Aplastic anaemia. The diagnosis was made according to standardised criteria following bone marrow aspiration and bone biopsy [18].

Intensive Care Unit (ICU). The patients were transferred to ICU in presence of (i) active bleeding; (ii) severe encephalopathy (grade 3- or coma); or (iii) the need for diagnostic or therapeutic procedures (e.g., liver biopsy, measurements of intracranial venous pressure; plasmapheresis).

Indications to LT. Progressive coagulopathy (INR > 4) and/or severe hepatic encephalopathy (grade III–IV or coma) were indications to be listed for LT.

### 2.3. Division into Two Groups and Statistics

Patients were divided into two groups, which corresponded to two eras:

- Group 1 (G1): patients diagnosed with ALF from 1997 to 2009.
- Group 2 (G2): patients diagnosed with ALF from 2010 to 2022.

Data on baseline features (which included data collected at the time of admission), need for LT and outcome were analysed and compared between the two groups.

Data are reported as medians and interquartile ranges. Continuous variables were compared by a Mann–Whitney U test. Categorical variables were compared by Pearson’s Chi-square test. We use Kaplan–Meier curves to report the transplant free-survival in both groups. A *p* value of 0.05 or less was assigned significance.

## 3. Results

Over a 26-year period, 90 children were diagnosed with ALF (M/F = 43/47) and enrolled in this study. All patients fulfilled PALF criteria for the diagnosis [1]. The median age at presentation was 4.6 years (IQR 1.2–10.4). The age distribution was: <1 month 7 patients (8%); 1 to 12 months 13 patients (14%); >1 year 70 patients (78%). Baseline features are reported in Table 1.

### 3.1. ALF Aetiology

*Autoimmune hepatitis*: 16 patients (18%, median age 9.6 years, IQR 5.3–13.9, M/F = 7/9) were diagnosed with AIH (*n* = 8 type 1 and *n* = 8 type 2). At our centre, all patients diagnosed with AIH were treated with IV steroids (methyl-prednisolone) [13,14]. The immunosuppressive treatment was well tolerated in all but 1 patient, a 17-year-old girl with AIH-1 who developed mood changes (HE was excluded) that were eventually solved when the dose of steroids was tapered.

*Paracetamol overdose*: 10 children (11%, M/F = 2/8, median age 2.7 years, IQR 1.2–3.7) had ALF due to paracetamol overdose; 1 of them was 9 months old, and the others were older than 1 year. In all cases, the parents inadvertently administered a toxic dose of the drug.

*Wilson disease*: 8 children (9%, M/F = 2/6, median age 12.4 years, IQR 11.7–13.7) were diagnosed with Wilson disease. Two had positive autoantibodies (ANA 1:640 in 1 patient; ANA:1.40 and ASMA 1:80 in 1) and high IgG.

*Inborn errors of metabolism (IEMs)*: 6 patients (7%, M/F = 4/2, median age 0.6 years, IQR 0.09–1.8) had a metabolic cause of ALF. Four patients were aged from 1 to 12 months. Parental consanguinity was reported in one case, and family history was positive for IEMs in one patient who had a brother who died at 21 days of life. The aetiologies were urea cycle disorders (ornithine transcarbamylase deficiency and HHH syndrome) in two children aged 26 and 30 months, respectively. Four patients were diagnosed with galactosaemia *n* = 1, glycogen storage disease *n* = 1 and mitochondrial respiratory chain defects (*n* = 2 new-born patients).

*Mushroom poisoning*: 6 male children (7%, M/F = 4/2, aged 9.3 years, IQR 5.5–13.3) had ALF caused by the ingestion of amanitin-containing mushrooms.

*Infections*: 5 children (6%, M/F = 4/1, aged 9.3 years, IQR 5.5–13.3) had ALF caused by viral infections, 3 patients had ALF from hepatitis A (HAV) and 1 from Parvovirus B19. One patient (2-year-old, with cystic fibrosis) had ALF triggered by EBV infection.

*Neonatal hemochromatosis* (NH): NH was diagnosed in 2 patients aged 15 days. Both were treated with high-dose intravenous immunoglobulin (plus exchange transfusion in 1). One patient (female) recovered with medical treatment, the second one (male) required LT. As of the last follow up, he is well (aged 10 years); this case has been described separately [19].

*Indeterminate ALF*: in 37 children (41%, M/F = 19/18, median age 1.9 years, IQR 0.5–7.5), the aetiology was unknown. Some 3 patients (11%) were younger than 1 month, 9 (35%) were infants, and 25 (67%) were older than 1 year.

**Figure 1:**
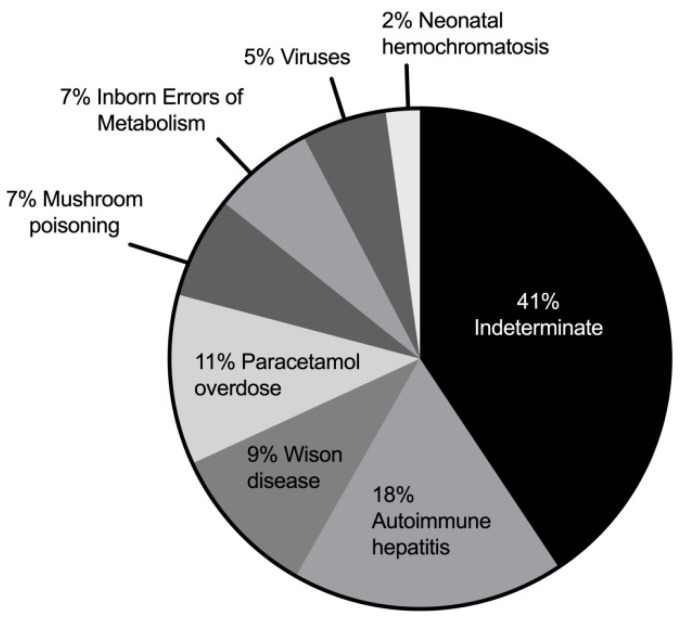
Aetiology distribution of a cohort of 90 Italian children presenting with acute liver failure.

The aetiology distribution including all patients is reported in Table 1 and Figure 1.

Comparing the two groups, the percentage of children diagnosed with ID-ALF tended to be lower in G2 compared to G1 (32% vs. 50% in G1, *p* = 0.09). In G2, the number of patients diagnosed with IEMs, WD, NH and viral infections (15/44, 34%) was significantly higher compared to G1 (6/46, 13%; *p* = 0.02).

### 3.2. Clinical Features and Laboratory Investigations

On admission, the majority of patients presented with jaundice (*n* = 48 patients, 53%) and gastrointestinal (GI) symptoms (including vomiting, diarrhoea and malaise; *n* = 45, 50%). Only 19 patients (21%) had fever. HE was recorded in 69 patients (77%; grade 1 = 3 pts, grade 2 = 16; grade 3 = 24; grade 4 = 26) and severe HE (grade 3 and 4) in 50 (55%) (Table 1).

At onset, laboratory features were (median values): ALT 2090 U/L (IQR 695–4426 U/L), total bilirubin 13.1 mg/dL (4.0–21.7 mg/dL), serum ammonia 134 µmol/L (66–195 µmol/L), international normalised ratio (INR) 3.4 (2.8–4.8).

Autoantibodies and IgG levels were tested in 90/90 (100%) and in 87/90 (97%) patients, respectively. Among patients with AIH, 100% had positive autoantibodies (16/16) and 81% high IgG levels (13/16).

Conversely, in children with ALF due to other aetiologies, autoantibodies were positive in 12/74 patients (16%; 6 patients with ID-ALF, 2 with WD, 3 with viral infection, 1 with paracetamol overdose). Histology was available in 59/90 patients (65%) as core needle biopsies (*n* = 27) or whole organs following transplantation (*n* = 32). Among 27 children who underwent liver biopsy (*n* = 9 with ID-ALF, *n* = 12 with AIH, *n* = 6 other aetiologies), none developed major complications. We found that massive/submassive necrosis was the most common histological feature. Cirrhosis was reported in 18 of 90 patients (20%; *n* = 5 with ID-ALF, *n* = 8 with AIH, *n* = 5 with WD; 10 from G1 and 8 from G2).

Comparing the two groups, gastrointestinal symptoms were more common in G2 compared to G1 (61% vs. 39%, *p* = 0.05). The median values of albumin and serum ammonia were lower in G2 compared to G1 (*p* = 0.009 and *p* = 0.01, respectively). Prevalence of positive autoantibodies tended to be higher in G1 (54% vs. 34% in G2, *p* = 0.06) although SMA and ANCA positivity was more common in G2 (*p* = 0.02 and *p* = 0.01). Ascites was more commonly diagnosed in G2 compared to G1 (34% vs. 6%, *p* = 0.001). There was no statistical difference in the other parameters (Table 1).

### 3.3. Management of PALF

On admission, a conservative management was started in all patients diagnosed with ALF to support the liver function regardless of the underlying aetiology. Furthermore, a specific medical treatment was started when a specific aetiology of ALF was identified; the protocol used at our centre has been reported previously [13,14].

In this cohort, 21 patients (23%) were treated with IV steroids (methyl-prednisolone 2 mg/kg/day; *n* = 9 patients in G1 and *n* = 12 in G2); 16 with AIH (3 received also cyclosporine) and 5 with ID-ALF (*n* = 4 in G2).

Among 16 patients with AIH (*n* = 8 patients in G1 and *n* = 8 in G2), the majority of them (9/16, 56%) improved and had a good transplant free survival, mainly in G2 (6/8 patients; 75%). Among 9 patients who recovered with medical therapy, 5 (5%) had severe HE (grade 3 or 4 in 4 patients and coma status in 1) but were rescued with steroid treatment without complications.

Among 5 patients with ID-ALF (*n* = 1 patient in G1 and *n* = 4 in G2), 3/5 patients recovered without requiring LT, mainly in G2 (3/4 patients; 75%).

In this cohort, 12 patients required some extracorporeal liver support treatment

(14%; *n* = 7 in G1 and *n* = 5 in G2); 10 received continuous renal replacement treatment (CRRT), and 8 received plasmapheresis (1 with ABO incompatible graft, 2 with Wilson disease, 3 with AIH, 2 with ID-ALF) (Table 2).

### 3.4. Outcome

**Figure 2:**
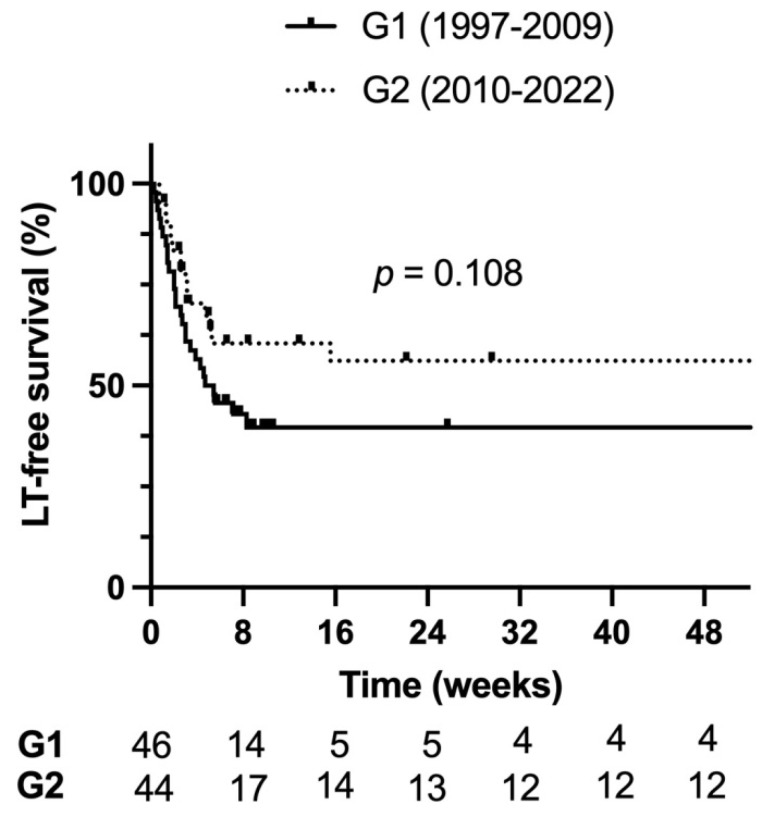
Kaplan–Meier native liver survival curve comparison of patients with ALF according to era. LT: liver transplant. G1: group 1. G2: group 2.

**Figure 3:**
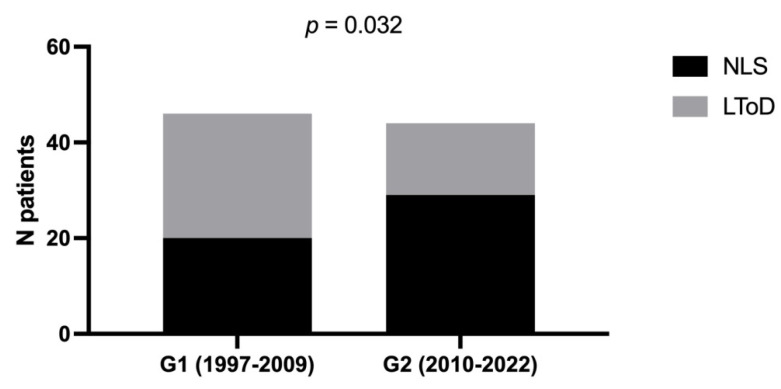
Comparison of the outcomes of patients with ALF according to era. LToD: liver transplant or death on waiting list. NLS: native liver survival; G1: group 1. G2: group 2.

**Figure 4:**
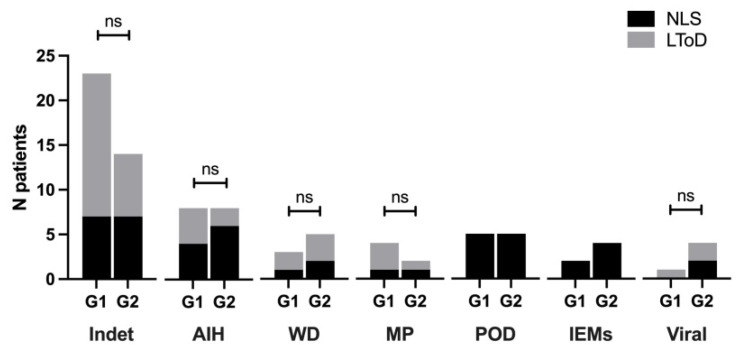
Comparison of the outcomes of patients with ALF according to era and aetiology. LToD: liver transplant or death on waiting list. NLS: native liver survival; G1: group 1. G2: group 2. AIH: autoimmune hepatitis; WD: Wilson disease; MP: mushroom poisoning; POD: paracetamol overdose; IEMs: inborn errors of metabolism; ns: not significant.

A total of 52 patients (58%) were listed for LT. Eight patients improved with medical treatment and were removed from the list (*n* = 6 in G2), 3 died on the waiting list (*n* = 1 in G2), and 41 (45%) underwent LT (median time on the waiting list was of 6 days, IQR, 2–11) (Table 2). Outcome according to aetiology is reported in Table 3. On KM analysis, the transplant-free survival was lower in G2 compared to G1, although this result did not reach statistical significance (Figure 2). Overall, the transplant rate was significantly lower in G2 (15/44, 34%) compared to G1 (26/46, 56%, *p* = 0.03) (Figure 3); no significant differences in the outcomes according to era and aetiology were reported (Figure 4).

A higher survival rate without transplant was seen in patients with ALF due to paracetamol overdose (10/10 patients, 100%) and metabolic disorders (6/6, 100%). Conversely, a higher need for LT was observed in children with WD (5/8, 62%) and ID-ALF (23/37, 62%), without differences between the two groups.

In this study, the median follow-up was 2 years (IQR, 0.4–4.7 years) in the entire cohort and 4.6 years (IQR 2.0–10.8 years) among the transplanted patients. The survival rate was of 95% at last follow up (G1 = 43/46, 93%; G2 = 42/44, 95%, *p* > 0.05). A total of 5 patients died, 3 of which were already mentioned above. The fourth patient (with AIH type II) died 2 years after LT due to septic shock. The fifth patient (with ID-ALF) after the transplant developed neurological sequelae and died 2 months later due to sepsis and multi-organ failure. In this patient, a mitochondrial disorder was suspected; however, extensive metabolic investigations were normal, including a muscle and skin biopsy.

Six patients (all in G2; M/F = 3/3; median age at diagnosis of ALF of 1.9 years, IQR 0.7–7.5) developed aplastic anaemia after a median time of 50 days (IQR 38–80) after the diagnosis of ID-ALF. Some 4 out of 6 had severe HE (grade 3 *n* = 3 and grade 4 *n* = 1). Of interest, they had a mean value of serum lymphocytes significantly lower than the other patients [946 × 10^3^/μL (±537) vs. 3079 × 10^3^/μL (±2760)]. Regarding the liver function, 4 patients recovered with medical therapy (3 were treated with IV steroids), whereas 2 patients (aged 4.8 and 1.7 years) required LT. According to standardised protocols, all patients were considered for allogeneic hematopoietic stem cell transplantation, which was available only in 1 patient. The other 5 patients were treated with immunosuppressive therapy; at last follow up they are alive and well [18].

Neurological complications were reported in two children, aged 2 and 20 months, respectively, one with cerebral atrophy and spastic tetraparesis and one with a developmental delay and epilepsy.

A 13-year-old boy with ALF due to AIH-2 was treated with steroids successfully and did not require LTX. Eventually, he developed idiopathic pulmonary hypertension which was successfully treated with sildenafil.

## 4. Discussion

In this study, we reported our experience in children diagnosed with ALF from 1997 to 2022. We divided the patients into two groups that reflected two eras, and compared them to see whether they differed. At presentation, all children had similar clinical and biochemical features as well as the same prevalence of HE.

A first finding of this study is that during the second period (G2 = 2010 to 2022), the percentage of patients diagnosed with ID-ALF was lower compared to the first period (G1 = 1997 to 2009). Consistent with this, the overall percentage of patients diagnosed with WD, IEMs, NH, or viral infection was significantly higher in G2 compared to G1.

These results likely suggest a trend of improvements in the diagnostic work up of children with ALF. In our experience, performing extensive laboratory investigations (in each child diagnosed with ALF) and considering liver biopsy as a helpful diagnostic tool when no aetiology is identified has surely contributed to improving the diagnostic accuracy in this cohort of patients, allowing us to provide more targeted management to these patients.

In this series, the prevalence of AIH in children older than 1 year was 23%, which is quite high compared with previous reports and the PALF study group (all reported a prevalence < 10% of cases) [20,21]. Identifying AIH as the cause of ALF is surely challenging. Studies have demonstrated that in many referral centres, autoantibodies are not routinely tested in ALF, and therefore some patients with AIH might end up with an incorrect diagnosis of ID-ALF [22,23]. Among 703 children recorded in the PALF database, only 55% received an extensive immunological profile, suggesting that AIH is likely underdiagnosed in this setting [20]. At our centre, all children with ALF undergo immunological investigations by 48 h after admission, including all relevant autoantibodies [ANA, SMA, LKM-1, anti-liver cytosol 1 (LC1), anti-soluble liver antigen, (SLA)]. This strategy has likely improved the diagnostic accuracy of AIH among our patients. In our cohort of patients, the identification of children with AIH allowed us to start immunosuppressive treatment promptly and rescue the native liver in a high percentage of cases, especially in the second era (Figure 4).

Liver biopsy, which was previously considered a contraindication in children with ALF, in our experience is a helpful diagnostic tool in selected cases in which the aetiology of ALF is uncertain (e.g., AIH, Wilson disease), or when a malignant infiltration is suspected [6,11,24]. In this cohort, one third of children (27/90) underwent liver biopsy without developing major complications. Of interest, 20% of patients had cirrhosis, confirming, as already published in our previous study, that a proportion of patients with PALF (mainly those with AIH, WD and indeterminate aetiology) have an underlying chronic condition that may present as acute liver failure [17].

Of interest, in G2, the prevalence of ascites was significantly higher compared to G1, despite cirrhosis not being statistically different between the two groups. This might be explained by lower albumin levels recorded in G2, which likely favoured fluid accumulation in this group of patients.

In Italy, in order to assist the clinicians in the management of children with ALF, recommendations on diagnostic approach of children with ALF, also referring to autoantibodies titers and indications for liver biopsy, have been published by Liver Disease working group of the Italian Society of Gastroenterology, Hepatology, and Nutrition (SIGENP) [6].

Another relevant finding of this work is that the percentage of children requiring LT was significantly lower during the second period (G2). Identifying the underlying aetiology is crucial to initiate disease-specific treatment in attempts to avoid a transplant. There are causes of ALF associated with an increased (e.g., paracetamol toxicity, hepatitis A) or reduced (e.g., WD or ID-ALF) likelihood of recovery after medical therapy [11,25]; therefore, recognizing the cause of ALF is paramount.

Our results demonstrate that diagnosing children with autoimmune ALF is important because they may respond to steroids successfully even in presence of severe HE. In this study, 56% of children with autoimmune ALF (63% of them with severe HE) recovered after steroid treatment, without requiring LT and without experiencing complications related to immunosuppressive treatment.

This confirms that AIH is a potentially reversible cause of ALF. Of interest, 5 children with ID-ALF were also treated with steroids, 3 of whom recovered without requiring LT. Similar results were reported in a paediatric study from the United States, in which 28 children with ID-ALF were treated with IV methyl-prednisolone (*n* = 20 at the dosage of 0.5–4 mg/kg/die; *n* = 8 at dosage of 10 mg/kg/die); 13 patients (46%) recovered with their native liver [26].

The satisfactory response to steroid treatment may be explained by the fact that ID-ALF in children is characterised by a dense CD8+ T-cell hepatic infiltrate that can be considered a biomarker of immune dysregulation. This suggests that some children with ID-ALF may benefit from immunosuppressive treatment [27].

Different results are reported in adults, in whom authors described only little benefit from steroid treatment in severe and fulminant forms of AIH, suggesting a different response to steroids between the adult and the paediatric population, which may be further explored in future studies [28].

With regard to the need for LT, we found that there were no differences between the two periods (G1 and G2). Our patients with indeterminate ALF required LT in 60% of cases, mainly in G1, and this percentage is similar to that reported by the PALF group [20].

In this cohort, the spontaneous recovery was more likely in children with ALF due to paracetamol overdose (10/10 pts, 100%) and IEMs (6/6 pts, 100%).

This suggests the importance of identifying the cause of ALF, because some aetiologies can be treated with medical therapy successfully.

In our patients, the survival rate without LT of patients with ALF is 55%, which is higher compared to previous studies [11,29]. Results from this study and from the PALF study group suggest that there have been some improvements in the medical management of children with ALF [13,14,25].

Of interest, in this cohort, 16% of patients with ID-ALF (all included in G2) developed AA by 90 days after the diagnosis of ALF. Aplastic anaemia was diagnosed by bone marrow biopsy in all (100%), and it was classified as severe AA according to the standard classification [18].

Hepatitis-associated aplastic anaemia (HAA) is a form of acquired AA in which bone marrow failure develops after an episode of acute hepatitis or liver failure. It usually develops by 8 weeks after the diagnosis of ALF, although in some cases, AA may occur simultaneously, or more rarely, before the diagnosis of hepatitis/ALF [30].

Pathogenetic mechanisms of HAA have not yet been identified, but an immune-mediated process that targets similar antigens in the liver and bone marrow cells is thought to be responsible. Indeed, immunosuppressive treatments (IST) with cyclosporine and antithymocyte globulin (ATG) and hematopoietic stem cell transplantation (HSCT) have been successfully used for its treatment [31]. Similarly, in this cohort, five out of six patients recovered with immunosuppressive treatment, and only one underwent bone marrow transplantation.

## 5. Conclusions

In conclusion, we report a lower rate of children classified as ID-ALF and a lower transplant rate in the more recent era.

This suggests that identifying the aetiology of ALF is crucial, because this may positively impact on outcome. In our experience, (i) performing extensive immunological and metabolic investigations; (ii) including liver biopsy as diagnostic tool; and (iii) utilizing steroids in children with AIH and ID-ALF have all likely contributed to improving the overall management of children with ALF.

## Figures and Tables

**Table 1:** Characteristics at baseline in 90 children with ALF divided into two groups.

Variables	All Patients *n* = 90	Group 1 (1997–2009) *n* = 46	Group 2 (2010–2022) *n* = 44	*p* Value
**Males, *n* (%)**	43 (48%)	25 (54%)	18 (41%)	0.214
**Age at onset, years**	4.6 (1.2–10.4)	3.1 (0.7–8.7)	5.0 (1.8–12.1)	0.124
**Aetiologies**				
Indeterminate	37 (41%)	23 (50%)	14 (32%)	0.09
AIH	16 (18%)	8 (17%)	8 (18%)	1.00
Type 1	8	3	5	-
Type 2	8	5	3	-
Wilson disease	8 (9%)	3 (7%)	5 (11%)	0.48
Neonatal hemochromatosis	2 (2%)	0	2 (5%)	0.23
Inborn errors of metabolisms	6 (7%)	2 (4%)	4 (9%)	0.42
Galactosemia	1	0	1	-
Urea cycle defect	2	1	1	-
Glycogen storage disease IV	1	0	1	-
Mitochondrial respiratory Chain defects	2	1	1	-
Mushroom poisoning	6 (7%)	4 (9%)	2 (5%)	0.67
Paracetamol	10 (11%)	5 (11%)	5 (11%)	1.00
Viral infection	5 (5%)	1 (2%)	4 (9%)	0.19
**Symptoms at onset**				
Fever	19 (21%)	11 (24%)	8 (18%)	0.60
GI symptoms	45 (50%)	18 (39%)	27 (61%)	**0.05**
Jaundice	48 (53%)	22 (48%)	26 (59%)	0.30
**Laboratory features**				
ALT, IU/L	2090 (695–4426)	2187 (801–4798)	2037 (354–3488)	0.08
INR	3.4 (2.8–4.8)	3.8 (2.9–4.8)	3.2 (2.4–4.8)	0.80
WBCs·10^3^/μL	8000 (5490–10,370)	8265 (5915–11,835)	7895 (5167–9662)	0.58
Lymphocytes·10^3^/μL	2250 (1425–3895)	2620 (1685–4625)	2040 (1180–3530)	0.08
Hb, g/dL	10 (9–12)	9.9 (8.8–11.7)	10.6 (9.1–11.5)	0.75
Platelets·10^3^/μL	181,000 (96,000–280,000)	193,500 (116,000–265,750)	163,500 (84,250–288,575)	0.87
Albumin, g/dL	3.3 (3–3.8)	3.3 (3.1–4)	3.1 (2.8–3.4)	**0.009**
Tot bilirubin, mg/dL	13.1 (4–21.7)	17.1 (6.5–23.5)	11.5 (3.4–19.1)	0.15
Ammonia, µmol/L	134 (66–195)	153 (99.5–220.5)	94 (58–145)	**0.01**
**Positive autoantibodies**	40 (44%)	25 (54%)	15 (34%)	0.06
ANA (≥1:20): *n* (%)	16 (18%)	6 (13%)	10 (23%)	0.27
SMA (≥1:20): *n* (%)	19 (21%)	5 (11%)	14 (32%)	**0.02**
Anti-LKM-1 (≥1:10): *n* (%)	7 (7%)	5 (11%)	2 (4%)	0.43
Anti-LC1: *n* (%)	5 (5%)	3 (6%)	2 (4%)	1.00
ANCA: *n* (%)	9 (10%)	1 (2%)	8 (18%)	**0.01**
IgG g/L (nv 5–18 g/L)	10.6 (8.4–15.1)	11.1 (8.2–15.1)	10.5 (8.6–14.1)	0.64
**Ascites, *n* (%)**	18 (20%)	3 (6%)	15 (34%)	**0.001**
Splenomegaly *, *n* (%)	25 (28%)	10 (22%)	15 (34%)	0.24
**HE grade III–IV, *n* (%)**	50 (55%)	29 (66%)	20 (45%)	0.13
**Liver histology (available in 59 patients) §**				
**Cirrhosis, *n* (%)**	18 (20%)	10 (22%)	8 (18%)	0.793

ALF: acute liver failure; AIH: autoimmune hepatitis; GI: gastrointestinal; ALT: alanine aminotransferase; WBCs: white blood cells; Hb: haemoglobin; ANA: antinuclear antibody; SMA: smooth muscle antibody; LKM-1: liver–kidney microsome antibody type 1; LC1: liver cytosol antibody type 1; HE: hepatic encephalopathy; *: detected on liver scan. §: as core needle biopsy in 27 and whole organs following transplantation in 32.

**Table 2:** Treatment and outcome in 90 children with ALF, comparison between two eras.

Variables	All Patients *n* = 90	Group 1 (1997–2009) *n* = 46	Group 2 (2010–2022) *n* = 44	*p* Value
Treatment with steroids, *n* (%) *	21 (23%)	9 (20%)	12 (27%)	ns
**Extracorporeal liver support, *n* (%)**	12 (13%)	7 (15%)	5 (11%)	ns
**Listed for transplant, *n* (%)**	52 (58%)	29 (63%)	23 (52%)	ns
Median time on waiting list (days)	6 (2–11)	6.5 (2–10)	5 (2–13)	ns
Died on waiting list	3	2	1	ns
Recovery without TX	8	2	6	ns
Transplanted	41	26	15	**0.03**
Split graft **	34	23	11	ns
Whole graft	7	3	4	ns
**Outcome at last FU**				
Survived	85 (94%)	43 (93%)	42 (95%)	ns
Died	5 (5%)	3 (6%)	2 (4%)	ns
**Complications after ALF**				
- Aplastic anemia	6 (7%)	0	6 (14%)	**0.01**
- Neurological sequaele	2 (2%)	2 (4%)	0	ns

* 5 patients with AIH were treated also with cyclosporine (*n* = 4 G1, *n* = 1 G2); **: 28 received the segments II–III (*n* = 19 in G1 and *n* = 9 in G2); 6 received the segments I-IV (*n* = 4 in G1 and *n* = 2 in G2).

**Table 3:** Outcome according to aetiology in 90 patients with acute liver failure, whole period and comparison between two eras.

Causes of ALF	Outcome Whole Period (1997–2022)				Survival			*p* Value
	Patients	Recovery without TX	LT	Died	Whole Period	G1 (1997–2009)	G2 (2010–2012)	G1 vs. G2
Autoimmune hepatitis	16	9	6	1 *	94%	87%	100%	ns
Wilson’s disease	8	3	5	0	100%	100%	100%	ns
Neonatal hemochromatosis	2	1	1	0	100%	-	100%	-
Metabolic disorders	6	6	0	0	100%	100%	100%	ns
Paracetamol overdose	10	10	0	0	100%	100%	100%	ns
Mushroom poisoning	6	4	2	0	100%	100%	100%	ns
Viral infection	5	2	2	1 *	80%	100%	75%	ns
Indeterminate	37	11	23	3 **	92%	91%	92%	ns
All aetiologies	90	44	41	5	94%	94%	95%	ns

ALF: acute liver failure; LT: liver transplantation; *: died on waiting list; **: 1 patient died on waiting list and 2 after LT.

## Data Availability

Data will be made available on request by the corresponding author.
